# Impact of the 7-bp deletion in *HvGA20ox2* gene on agronomic important traits in barley (*Hordeum vulgare* L.)

**DOI:** 10.1186/s12870-017-1121-4

**Published:** 2017-11-14

**Authors:** Serafima Teplyakova, Marina Lebedeva, Nadezhda Ivanova, Valentina Horeva, Nina Voytsutskaya, Olga Kovaleva, Elena Potokina

**Affiliations:** 1 Saint Petersburg State University, Universitetskaya emb.7/9, St. Petersburg, 199034 Russia; 2 N.I. Vavilov Institute of Plant Genetic Resources (VIR), Bolshaya Morskaya, 42-44, 190000 St. Petersburg, Russia; 3 Saint Petersburg State Forest Technical University, Institutskiy per, 5, 194021 St. Petersburg, Russia

**Keywords:** Semi-dwarf barley, *sdw1/denso*, Functional polymorphism, QTL, Heading date, Plant height, Grain protein content, Thousand grain weight

## Abstract

**Background:**

Alike to Reduced height-1 (*Rht*-1) genes in wheat and the semi dwarfing (*sd-1*) gene in rice, the *sdw1/denso* locus involved in the metabolism of the GA, was designated as the ‘Green Revolution’ gene in barley. The recent molecular characterization of the candidate gene *HvGA20ox2* for *sdw1/denso* locus allows to estimate the impact of the functional polymorphism of this gene on the variation of agronomically important traits in barley.

**Results:**

We investigated the effect of the 7-bp deletion in exon 1 of *HvGA20ox2* gene (*sdw1.d* mutation) on the variation of yield-related and malting quality traits in the population of DHLs derived from cross of medium tall barley Morex and semi-dwarf barley Barke. Segregation of plant height, flowering time, thousand grain weight, grain protein content and grain starch was evaluated in two diverse environments separated from one another by 15° of latitude. The 7-bp deletion in *HvGA20ox2* gene reduced plant height by approximately 13 cm and delayed flowering time by 3–5 days in the barley segregating DHLs population independently on environmental cue. On other hand, the *sdw1.d* mutation did not affect significantly either grain quality traits (protein and starch content) or thousand grain weight.

**Conclusions:**

The beneficial effect of the *sdw1.d* allele could be associated in barley with lodging resistance and extended period of vegetative growth allowing to accumulate additional biomass that supports higher yield in certain environments. However, no direct effect of the *sdw1.d* mutation on thousand grain weight or grain quality traits in barley was detected.

**Electronic supplementary material:**

The online version of this article (10.1186/s12870-017-1121-4) contains supplementary material, which is available to authorized users.

## Background

The tremendous success of the “Green Revolution” of the XX century was achieved due to the introduction in agriculture the new cereal varieties distinguished by a shortened thick stem, resistant to lodging. These varieties made it possible to change agricultural technology, to increase the dose of mineral fertilizers, and, as a result, dramatically improved the productivity of cereal crops. For wheat and rice the “Green Revolution” genes were involved in a gibberellic acid (GA) metabolism influencing plant height (dwarfing), lodging resistance, and, consequently, harvest index (e.g. [1]). In hexaploid wheat, dwarfing has been achieved mainly through the introduction of the mutant alleles of Reduced height-1 (*Rht*-1) genes that encode DELLA proteins, which act to repress GA-responsive growth [2]. In rice, the record yields obtained in the 1960s throughout Asia were owing to semi-dwarfing *sd-1* mutants carrying a deletion of 280 bp within the coding region of *Os20ox2* gene resulting in the nonfunctional 20-oxidase GA biosynthetic enzyme [3]. Thus, the genes involved in the metabolism of GA (semi dwarfing *sd-1* gene in rice and *Rht* gene in wheat) have played the crucial role in plant height reduction, which led to a significant increase in yields of the two major cereal crops. In barley, however, the impact of the semi-dwarfing genes on agronomical success of modern varieties still remains uncertain.

Two dwarfing genes, *sdw1/denso* and *uzu1.a* involved, respectively, in the metabolism of the GA and brassinosteroid hormones, were designated as “the Green Revolution” genes in barley [4]. Among them, the *sdw1* barley gene is an ortholog of the semi-dwarf *sd-1* gene in rice; the corresponding candidate gene *HvGA20ox2* encoding the gibberellin 20-oxidase was recently reported [5, 6]. Four mutant alleles of *sdw1* gene are known in barley. One of them *sdw1.c* (originally named *denso*) was found in a spontaneous mutant selected from the *cv*. Abed Denso in Denmark. Three other *sdw1* alleles have been obtained artificially with the use of mutagens: 1) *sdw1.a* mutant (originally named *sdw1*) were induced by X-rays in the Norwegian six-rowed barley *cv*. Jotun. 2) the *sdw1.d* mutant was obtained using X-rays treatment of cultivar Valticky and released in 1965 as *cv.* Diamant. Today cultivars with the *sdw1.d* mutation from Diamant are often described as «*sdw1/denso*». 3) *sdw1.e* named as ‘Risø no. 9265’ was isolated in a M2 generation from the variety Bomi treated by neutrons [7].

A pronounced success was achieved in molecular characterization of the *sdw1* barley alleles [5–8]. First, *HvGA20ox2* sequences of 2413 bp were compared between medium tall barley variety AC Metcalfe and semi-dwarf variety Baudin possessing the semi-dwarfing gene (*sdw1.d*); the A/G substitution was identified in the intron 2 co-segregating with plant height in AC Metcalfe/ Baudin DH progeny [5]. Next, the 7-bp deletion in exon 1 resulting in coding frame shifts of *HvGA20ox2* gene was suggested as the functional polymorphism of the *sdw1.d* allele from Diamant [8]. A complete deletion of *HvGA20ox2* gene was identified in semi-dwarf mutant Riso no. 9265 carrying *sdw1.e* allele, originated from the variety Bomi [7]. Five different sequence variations were identified by comparing the *HvGA20ox2* gene sequence of the *sdw1.c* mutant (Abed Denso) with the tall barley cultivars: they include a 1-bp deletion and a 4-bp insertion in the 5′ untranslated region as well as two synonymous mutations in coding sequence of *HvGA20ox2* gene [8]. While plant height variation was significantly associated with the 7-bp deletion of *HvGA20ox2* gene (*sdw1.d* allele) in a bi-parental mapping population and a natural population of barley varieties, none of the exposed sequence variations the *sdw1.c* mutants could explain dwarf phenotype [8].

All the *sdw1* barley mutants are distinguished by shorter and stronger culms supporting spikes and preventing lodging. However, the mutations in *HvGA20ox2* gene affect biosynthesis of the GA that control many processes in different plant tissues, thus, their pleiotropic effects also cause a number of unwanted agronomic traits such as reduction of grain size [4]. Both beneficial and deleterious effects associated with *sdw1* gene were reported for barley (reviewed in [9]). Recent success in molecular characterization of the *sdw1* alleles facilitated further studies focusing on the better understanding of their pleiotropic effect on agronomic performance of barley varieties.

In the present paper we estimated the effect causing by the 7-bp deletion in exon 1 of *HvGA20ox2* gene (the *sdw1/denso* allele) on the variation of economically important traits in the segregating population of the double haploid lines (DHLs) derived from the cross of the tall barley Morex and the semi-dwarf *sdw1.d* mutant cv. Barke. Barke inherited the *sdw1/denso* allele from Triumph via cv. Alexis (https://triticeaetoolbox.org/barley/), while Triumph is a descendant of cv. Diamant. We estimated the phenotypic effect of the *sdw1/denso* allele in two very diverse environments by the QTL mapping approach. Variation of plant height, heading date, thousand grain weight, grain protein and starch content in seeds was evaluated.

## Methods

### Plant material and field evaluation

Seeds of double haploid lines (DHLs) derived from the cross between Morex and Barke were kindly offered for the field evaluation by Nils Stein (Gatersleben, Germany). The DHLs population is abbreviated further in the text as Morex/Barke DHLs population.

Field trials of the Morex/Barke DHLs population were conducted in two experimental stations of VIR: in Pushkin (59°53′39 ″N, St. Petersburg region, North of Russia) and Krasnodar (45°02′55 ″N, South of Russia). The field experiment consisted of two replicate plots each containing a row of 50 plants of each doubled haploid line and two rows of each parent. Rows were spaced approximately 20 cm apart.

DHLs were evaluated for heading date (HD) that was calculated as number of days from sowing until first awns visible. Plant height (PH) was measured at full maturity: height to top of spike excluding awns was recorded for ten individuals per each DH line. PH records for Morex/Barke DHLs were obtained in 2015 (Pushkin).

Thousand grain weight (TGW) was measured at the conditioning humidity, according to standard protocols [10]: from the sample of grains for each DH line two weights were singled out, each with 500 seeds. The samples were weighed on the laboratory balances with an accuracy of 0.01 g. The sum of the results of weighing of two samples of 500 seeds was calculated.

Grain Protein Content (GPC) and starch content in dry seeds were analyzed using Infratec 1241 Grain Analyser (Foss, USA). The data to construct the calibration curve for GPC were obtained with Kjeltec Auto 1030 Analyzer (Sweden) by Kjeldahl method. The calibration curve for starch determination was developed with Evers’ Polarimetric Method.

### QTL mapping and statistical analysis

For the Morex/Barke segregating population a comprehensive genetic map was available consisting of 1068 SNP markers for 93 DHLs [11], the published genotyping data were used for the QTL analysis. Mapping of QTL was carried out with the Windows QTL Cartographer version 2.5 software [12] using the CIM algorithm (Composite Interval Mapping). The minimum threshold of LOD, significant at 95% (*p* = 0.05), was calculated based on the results of 1000 permutations. To visualize LOD scans a Perl script was used (available upon request).

### CAPS assay to reveal 7 bp deletion in exon 1 of HvGA20ox2 gene

PCR was performed with designed primers: forward 5′-CTCCCTCCCTCCCCGATTAC and reverse 5′-CCGGACACCTGGAAGAACCC. The reaction mix contained 1 × Taq-buffer (2.5 mM Mg2+); 200 μM dNTP; 2,5 U Tag DNA polymerase (Sileks, Moscow); 0.4 μM of each primer; ~ 30 ng of template DNA, 5% DMSO and sterile distilled water in a final volume of 25 μl. PCR cycling conditions consisted of an initial denaturation step of 95 °C for 3 min, followed by 30 cycles of 95 °C for 20 s, 63 °C for 30 s, 72 °C for 45 s, and a final extension cycle at 72 °C for 5 min. PCR products were detected on 1,3% agarose gel in TBE buffer, then were purified with ISOLATE II PCR and Gel Kit (Bioline) and sequenced in two ends (Eurogen, Moscow) using forward and reverse primers. The DNA sequences were aligned with Unipro UGENE software [13]. GenBank sequence accessions are: ‘Barke’ KX611232, ‘Triumph’ KX611233, ‘Morex’ KX611234, ‘Franklin’ KX789375.

To reveal the 7 bp deletion in exon 1 of *HvGA20ox2* gene 3 μl of the PCR product were digested with 1 U of *Hinf I* (Sibenzyme, Novosibirsk) in a total reaction volume of 15 μl containing 1х SEbuffer O (pH 7.6) for 2 h at 37 °C, followed by electrophoretic separation in 1.5% agarose gel in TBE buffer.

## Results

### Re-sequencing and mapping of HvGA20ox2 gene in Morex/Barke DHLs population

**Fig. 1:**
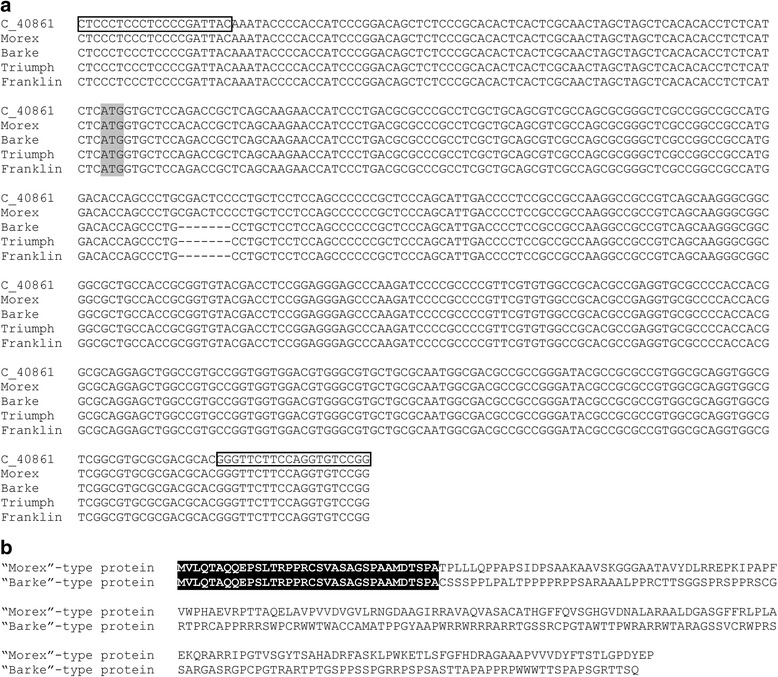
7-bp deletion detected in exon 1 of HvGA20ox2 in Barke (KX611232), Triumph (KX611233) and Franklin (KX789375) semi dwarf barley. Medium tall barley Morex (KX611234) has the intact exon 1. Numbers in parenthesis are GenBank accession numbers. **a** – multiple alignments include morex_contig_40861 (abbreviated as C_40861) that contains the coding sequence of HvGA20ox2 gene (MLOC_56462.1). The start codon is highlighted in *gray*. Primers positions are indicated by *boxes*. **b** – the predicted protein sequence resulting from 7 bp deletion in exon 1 of HvGA20ox2. The *black box* indicates 34 amino acids that are in common between intact and altered protein sequence

To prove that Barke is the *sdw1.d* mutant we re-sequenced the exon 1 of *HvGA20ox2* gene for the barley variety. To design primers we aligned the morex_contig_40861 (http://webblast.ipk-gatersleben.de/barley/viroblast.php) containing the published fragment of *HvGA20ox2* sequence and MLOC_56462.1 that is the coding sequence for *HvGA20ox2* gene (Fig. 1a). The PCR fragment of 488 bp was successfully amplified and sequenced for Barke, Triumph and Franklin barley varieties which are derived from the barley ‘Diamant family’ carrying semi-dwarf *sdw1*/*denso* allele, and also for Morex (tall barley). Deletion of 7 bp leading to the shift of reading frame in Barke, Triumph and Franklin was confirmed in exon 1 of *HvGA20ox2*. The intact and altered predicted proteins have just 34 amino acid sequence in common, followed by completely different polypeptides (Fig. 1b).

**Fig. 2:**
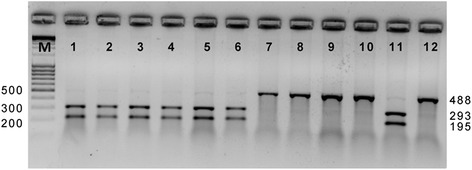
Genotyping of DHLs derived from Morex and Barke cross with CAPS marker to detect DHLs with the 7-bp deletion in exon 1 of *HvGA20ox2* gene (*sdw1/denso* allele, undigested fragment of 488 bp). DHLs with the intact exon 1 (Morex allele): lines 1–6, 11. DHLs with *sdw1/denso* allele: lines 7–10, 12. M - DNA size marker

To map the *HvGA20ox2* gene we developed the gene specific CAPS marker. The CAPS marker exposes whether a DH line has the 7-bp deletion (Barke allele) or carries intact exon 1 (Morex allele) (Fig. 2). Next, 94 DHLs from the cross of Morex and Barke were assessed with the gene-specific CAPS marker. As the result, the *HvGA20ox2* gene was mapped in the Morex/Barke DHLs population on the long arm of 3H chromosome in the position of 132.7 cM, co-segregating with SNP 1_0754 (Additional file 1).

## 7-bp deletion in exon 1 of *HvGA20ox2* associates with plant height and heading date segregation in Morex/Barke DHLs population

### Segregation of plant height in Morex/Barke DHLs population

**Fig. 3:**
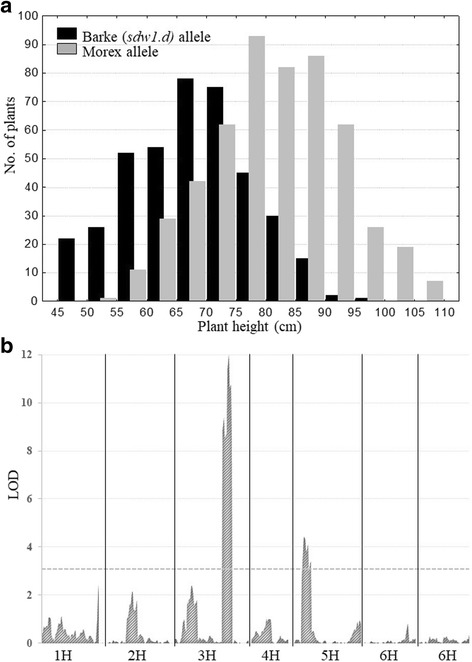
Influence of *sdw1.d* allele on plant height segregation in Morex/Barke barley mapping populations. **a** – bimodal distribution of plant height observed among the progeny of Morex/Barke *cross*; *black bars* correspond to the DHLs with 7-bp deletion in exon 1 of *HvGA20ox2*. **b** – LOD scans of Composite Interval Mapping (CIM) for plant height in Morex/Barke population in Pushkin (59°59′39″ N) in 2015. Barley chromosomes are designated 1H through 7H. *HvGA20ox2* gene-specific CAPS is the closest marker to the QTL on 3H

To verify whether the *sdw1.d* mutation is associated with the plant height segregation observed in the Morex/Barke DHLs population, the height of 920 plants (ten individual plants per each of 90 DHLs and two parents (Additional file 2) were compared considering the 7-bp deletion in exon 1 of the *HvGA20ox2* gene. The resulting bimodal distribution is shown in Fig. 3a. Two distinct peaks reflect significant difference between plant height values of the two allele classes (*p* < 0.0000001). For DH lines with Barke *sdw1.d* allele the averaged plant height varied from 50 to 87 cm with a mean value of 70 cm; for DH lines with Morex allele plant height ranged from 60 to 107, an average 83 cm. It was previously reported that the *sdw1* and *denso* allele reduced height by 10 to 20 cm [5, 14, 15].

The plant height variation was also subjected to QTL mapping. As the result, the highly significant QTL peak (LOD = 10.6, *p* < 0.05) was mapped on 3H in Morex/Barke DHLs population, co-segregating with the *HvGA20ox2* gene specific CAPS marker and explaining 31% of observed phenotypical variation (Fig. 3b).

### Segregation of heading date in Morex/Barke DHLs population

The Morex/Barke DHLs population was evaluated for heading date segregation in five environments: three years (2011–2013) in Pushkin and two years (2012, 2013) in Krasnodar. The two geographical locations are separated from one another by 15° of latitude; the day length on the date of seedlings emergence in 2012 was 17 h 30 min and 13 h 24 min respectively. The averaged heading date of the DH lines in the short day conditions (Krasnodar) was 12–20 days longer than in long day conditions (Pushkin) depending on the year (Additional file 3). The remarkable difference in flowering time was hardly due to alleles of *Ppd* genes, since for both Morex and Barke a recessive allele of *Ppd-H1* gene was reported, suggesting reduced response to the long day [16].

**Fig. 4:**
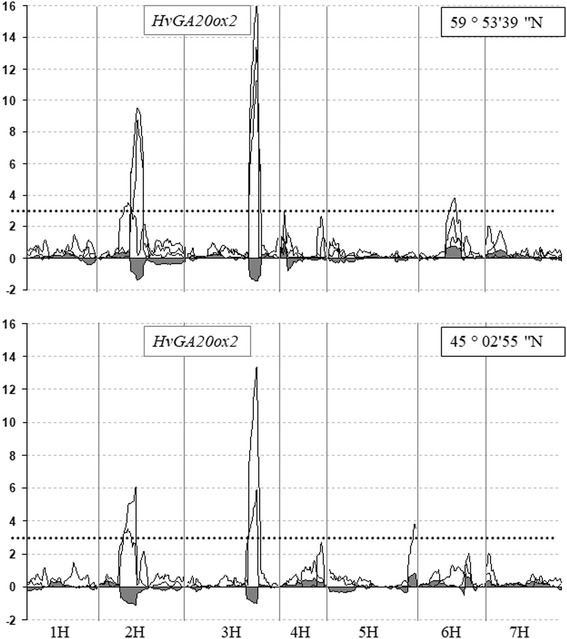
LOD scans of Composite Interval Mapping (CIM) for days to heading in 90 DH progeny lines derived from the Barke and Morex cross in two geographical locations: Pushkin (59°59′39″ N) in 2011, 2012, 2013 and Krasnodar (45°02′55″N) in 2011, 2013. Additive effect is shown by the gray fill path along the X axis. Barley chromosomes are designated 1H through 7H. *HvGA20ox2* gene-specific CAPS is the closest marker to the QTL on 3H in all environments tested

Two significant QTLs affecting heading date in Morex/Barke DH population were mapped on 2H and 3H chromosomes (Fig.4) in two different geographical locations. For both mapped QTLs the prolonged heading date was associated with Barke alleles. One of the QTLs was consistently mapped on the long arm of 3H in the position of 132.7 cM in all environments tested. The position of the QTL perfectly co-segregated with the *HvGA20ox2* gene-specific CAPS marker on Morex/Barke genetic map. The maximal proportion of variance (*R* ^*2*^) explained by the QTL varied from 28% to 41% in two geographical locations. DHLs, inheriting *sdw1/denso* allele from Barke, initiated flowering 3–5 days later than DHLs carrying the *HvGA20ox2* allele from Morex.

The closest SNP marker to the QTL on 3H in all five environments was SNP 1_0754; this SNP was co-segregating with BOPA SNP marker 11_0977 in OWB barley genetic map [11]. The SNP 11_0977, in turn, was mapped in the same position as a major QTL peak for heading date and plant height detected on 3H (139 cM) in SBCC145 x Beatrix DH population and was proposed to be the closest marker to the *sdw1/denso* dwarfing gene [17].

## Variation of thousand grain weight (TGW) and grain quality traits in Morex/Barke DHLs population depending on 7-bp deletion in exon 1 of *HvGA20ox2* gene

**Fig. 5:**
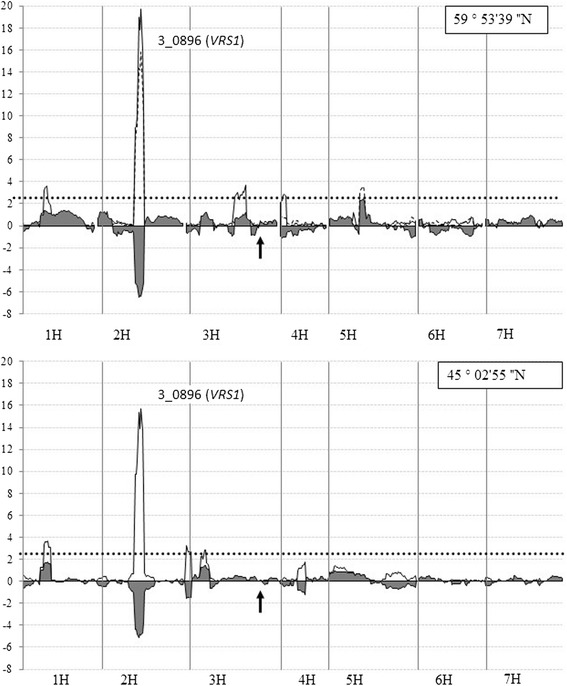
LOD scans of Composite Interval Mapping (CIM) of thousand grain weight in Morex/Barke barley population in three geographical locations: Pushkin (59°59′39″ N) in 2011, 2013, and Krasnodar (45°02′55″ N) in 2011. Additive effect is shown by the gray fill path along the X axis. SNP 3_0896 (*VRS1*) is the closest marker to the QTL on 2H in all environments tested. The position of the *sdw1/denso* gene is marked by the *black arrows*

TGW variation was estimated in the Morex/Barke DHLs population in three environments: Pushkin_2011, Pushkin_2013, Krasnodar_2011. The robust significant QTL peak for the trait was consistently mapped on 2H chromosome (77.6 cM) (Fig. 5), co-segregating with SNP 3_0896, which was reported as POPA SNP marker of the *VRS1* locus controlling the inflorescence type [18]. The allelic state of at the *VRS1* locus differentiates the wild type two-rowed barley and six-rowed barley. Loss-of-function mutations in the *HvHOX1* gene that underlies the *VRS1* locus cause a cessation of suppression of lateral-spikelet development and thus lead to the recessive six-rowed phenotype [19]. In the Morex/Barke cross the two-rowed parent Barke carries the dominant *Vrs1.b3* allele, while six-rowed parent Morex has the recessive *vrs1.a1* allele [18]. Polymorphism in the *VRS1* locus explained up to 48% of the TGW variation observed among the DHLs derived from the cross between Morex and Barke (Additional file 4). The two-rowed DHL offspring showed higher TGW values compared to six-rowed DHLs. However, no impact of the *sdw1/denso* locus on the trait variation was detected.

Grain protein concentration (GPC) is a primary determinant of grain value and malting quality in barley. While very low (< 90 g kg^−1^) grain protein concentration reduces the value of malting barley, high grain protein concentration (> 145 g kg^−1^) is associated with low levels of malt extract and increased likelihood of chill haze in finished beer [20]. Since grain protein concentration is impacted both by environmental conditions and genetic background, it was suggested as an ideal subject for QTL analysis and potential marker assisted selection. Starch content affects water uptake during steeping of barley and is considered as the important trait for malting industry [21]. To check whether *sdw1.d* allele affects GPC and starch content the two traits were assessed in Morex/Barke DHLs populations in five and three environments respectively. Alike to TGW analysis results, the 7-bp deletion in exon 1 of *HvGA20ox2* gene did not affected accumulation of protein and starch in grains, while significant influence of *VRS1* locus on GPC in Morex/Barke DHLs population was discovered in three out of five environments tested (Additional file 4).

## Discussion

In the present study we investigated effect of *sdw1/denso* allele on the variation of yield-related and malting quality traits in the population of DHLs derived from cross of medium tall barley Morex and semi-dwarf barley Barke. The latter belongs to the ‘Diamant family’ of the barley varieties inheriting the *sdw1/denso* allele from X-ray mutant cv. Diamant. The *sdw1/denso* allele has been reported for more than 150 new successful malting barley cultivars in Europe [22] and also has gained great acceptance in malting barley breeding programs around the world. The question was whether only the short stature of the *sdw1/denso* mutants and their resistance to lodging was the cause for the apparent agronomical success of semi-dwarfing barleys, or the pleiotropic effect of *sdw1/denso* allele on the yield-related traits has played the decisive role. For wheat it was previously reported that the direct effect of the *Rht* mutations is reduced plant height, but there is also an important pleiotropic effect causing increased assimilate partitioning to developing ears and an increased number of grains per spike and resulting in enhanced yield (reviewed in [4]). On other hand, the agronomic success of *sd-1* mutants in rice may be intimately linked to the developmental timing of stem growth and panicle development [3].

The *sdw1/denso* locus was mapped on 3H chromosome of barley few decades ago [23], since that time many QTL mapping studies reported the linkage of the *sdw1/denso* locus to agronomical important traits (reviewed in [9]). However, the candidate *HvGA20ox2* gene for *sdw1/denso* locus and the functional polymorphism assigned to the semi-dwarf alleles were defined just recently [5, 8]. The *sdw1/denso* allele is most likely resulted from the deletion of 7 bp in exon 1 of *HvGA20ox2* gene [8]. The deletion leads to the shift of reading frame of *HvGA20ox2* gene and may affect dramatically the structure of corresponding protein. *HvGA20ox2* encodes GA 20-oxidase that is involved in the final steps of gibberellin biosynthesis. Mutations of the *HvGA20ox2* lead to reduced endogenous GAs concentration affecting stem elongation and also flowering time, since GA acts a particularly important developmental switch between vegetative and reproductive development [24]. Corresponding to these reports, we detected the significant association between the 7-bp deletion in exon 1 of *HvGA20ox2* gene and the segregation of plant height and heading date in Morex/Barke DHLs population: DHLs inheriting the *sdw1/denso* allele from Barke were on average 13 cm shorter in height and initiated flowering 3–5 days later than DHLs carrying the intact *HvGA20ox2* allele from Morex. This also confirms previous observations that the *sdw1* barley mutants show a 10–20 cm reduction of plant height and 3 days heading delay [15, 25]. Besides, Maurer et al. [26] recently highlighted the important role of *sdw1/denso* gene in a whole plant’s life cycle: introgression of the wild *sdw1* allele into Barke genetic background from wild barley (*Hordeum vulgare ssp. spontaneum*) increased plant height by up to 12.3 cm, reduced the time required to reach shooting, flowering and maturity by 5.7, 4.3 and 4.0 days, respectively.

While the main characteristics of the *sdw1/denso* phenotype, such as a reduction of culm internode length, increased lodging resistance and delayed flowering are generally recognized, its effect on yield components or malting quality traits in barley is still questionable. QTLs for heading date, growth habit, yield, development score, plumpness and hectolitre weight were co-located with the *sdw1/denso* on the long arm of chromosome 3H (e.g. [6, 25]). Both positive and negative effects of *sdw1/denso* gene on the yield components in barley were reported [9, 22]. For example, DHLs derived from the cross of the barley variety Magnum and the variety Goldmarker (carrying the *sdw1/denso* gene) showed lower plant grain weights and 50 grain weights [27]. Grain yield of semi-dwarf lines derived from the crosses of semi-dwarf ‘Royal’ parent and eight tall barley lines was not significantly increased in all the cases [15]. The major issue reported for *sdw1/denso* barley varieties is the decreased thousand grain weight: replacement of *sdw1/denso* Barke alleles by those introgressed from wild barley increased TGW by up to 4.5 g [26]. On other hand, the *sdw1/denso* locus had a large positive effect on grain yield in DHLs population derived from the cross of medium tall AC Metcalfe and semi dwarf Baudin, explaining 49% of the variation [6].

Final grain yield in wheat and barley depends on grain number and grain weight (reviewed in [4]). The yield components influencing grain number include number of tillers bearing fertile spikes, extension of vegetative as well as reproductive growth, inflorescence architecture, culm hardiness, spike initiation, elongation and branching as well as spikelet formation. In the Morex/Barke segregating population the variation of grain weight (TGW) and grain quality (GPC) were toughly determined by the *VRS1* locus, differentiating the two-rowed and six-rowed DHLs progenies. No impact of the *sdw1/denso* locus on the yield component and grain quality traits was detected.

The ortholog of *HvGA20ox2* gene in rice genome, *sd1* is recognized as the classic Green Revolution gene. Similar to barley, large deletions in coding part of the corresponding rice gene *Os20ox-2* were discovered for rice semi-dwarf cultivars. Some of them (i.e. IR8) brought the Green Revolution to many countries in Asia [22] and still show the best agronomic performance [28]. In barley, however, no significant positive pleiotropic effect of *sdw1/denso* gene on the yield and quality components such as TWG or GPC was detected neither in the present study, nor in the most of previous reports. Though, an indirect positive effect of semi dwarfism on agronomic performance in barley could be assumed. Semi-dwarf barleys are more resistant to lodging than tall plants; lodging reduces not only barley yield and grain quality but also affects malt quality since the grain from lodged plants is often lighter in weight and lower in malt extract [29]. Compared to its ancestor cv. Valticky, the *sdw1.d* mutant cv. Diamant was reported to increase grain yields by 12% [30]. Triumph derived from Diamant is known as the best genetic sources for malting barley in Europe.

The positive or negative effect of *sdw1/denso* gene on the yield components in barley could also depend on different environments. Two-rowed *sdw1/denso* spring barleys could be highly advantageous in Western Europe with the long growing season allowing to accumulate additional biomass that supports higher yields. In hot and dry areas, however, the delay in heading date raises a risk that grains filling will occur concurrently with a drought and high summer temperatures which may harm yield performance of *denso*-carrying spring barley varieties. As a consequence, lateness in heading associated with *denso* was reported as another issue of barley breeding in Spain [17].

## Conclusions

In our study the 7-bp deletion in *HvGA20ox2* gene, which was recently proposed as the functional polymorphism of *sdw1/denso* locus in barley, was significantly associated with reduced plant height and delayed flowering time in the segregating population of DHLs derived from cross of medium tall barley Morex and semi-dwarf *sdw1.d* (*sdw1/denso*) variety Barke, independently on environmental cue. On other hand, the *sdw1.d* mutation did not affect either thousand grain weight or grain quality traits variation in this DHLs population. Thus, the beneficial effect of the semi dwarf *sdw1.d* allele in barley seems does not relate directly to the grain yield potential, but is associated with lodging resistance. Besides, in certain ecological environments the extended period of vegetative growth allows to accumulate additional biomass supporting higher grain yield.

## Additional files

Additional file 1: Mapping of the *HvGA20ox2* gene in the Morex/Barke DHLs population on the long arm of 3H chromosome and phenotypic trait data scored for DHLs of the Morex/Barke barley mapping population. (XLSX 27 kb)

Additional file 2: Plant height segregation evaluated for 10 individual plants per each of 90 DHLs and two parents the Morex/Barke DHLs population. (XLSX 15 kb)

Additional file 3: Flowering time (days to awn emergence) of 90 DHLs derived from Barke and Morex cross in 2011–2013 in two geographical locations: Pushkin (59° 53′ 39″ N) and Krasnodar (45°02′55″ N). (PPTX 72 kb)

Additional file 4: LOD scans, additive effect and percent of explained phenotypic variation (R2) of Composite Interval Mapping (CIM) performed for agronomic traits scored for DHLs of Morex/Barke barley mapping population. Obtained from output of Windows QTL Cartographer version 2.5 via Perl script. (XLSX 284 kb)
