# Supplementary material for: Impact of the 7-bp deletion in HvGA20ox2 gene on agronomic important traits in barley (Hordeum vulgare L.)
Source: BMC Plant Biol. 2017 Nov 14;17(Suppl 1):181. doi: 10.1186/s12870-017-1121-4 (PMC5688404; doi:10.1186/s12870-017-1121-4)
Supplement: Supplementary file 3 — Flowering time (days to awn emergence) of 90 DHLs derived from Barke and Morex cross in 2011–2013 in two geographical locations: Pushkin (59° 53′ 39″ N) and Krasnodar (45°02′55″ N). (PPTX 72 kb) [file 12870_2017_1121_MOESM3_ESM.pptx]

## Slide 1
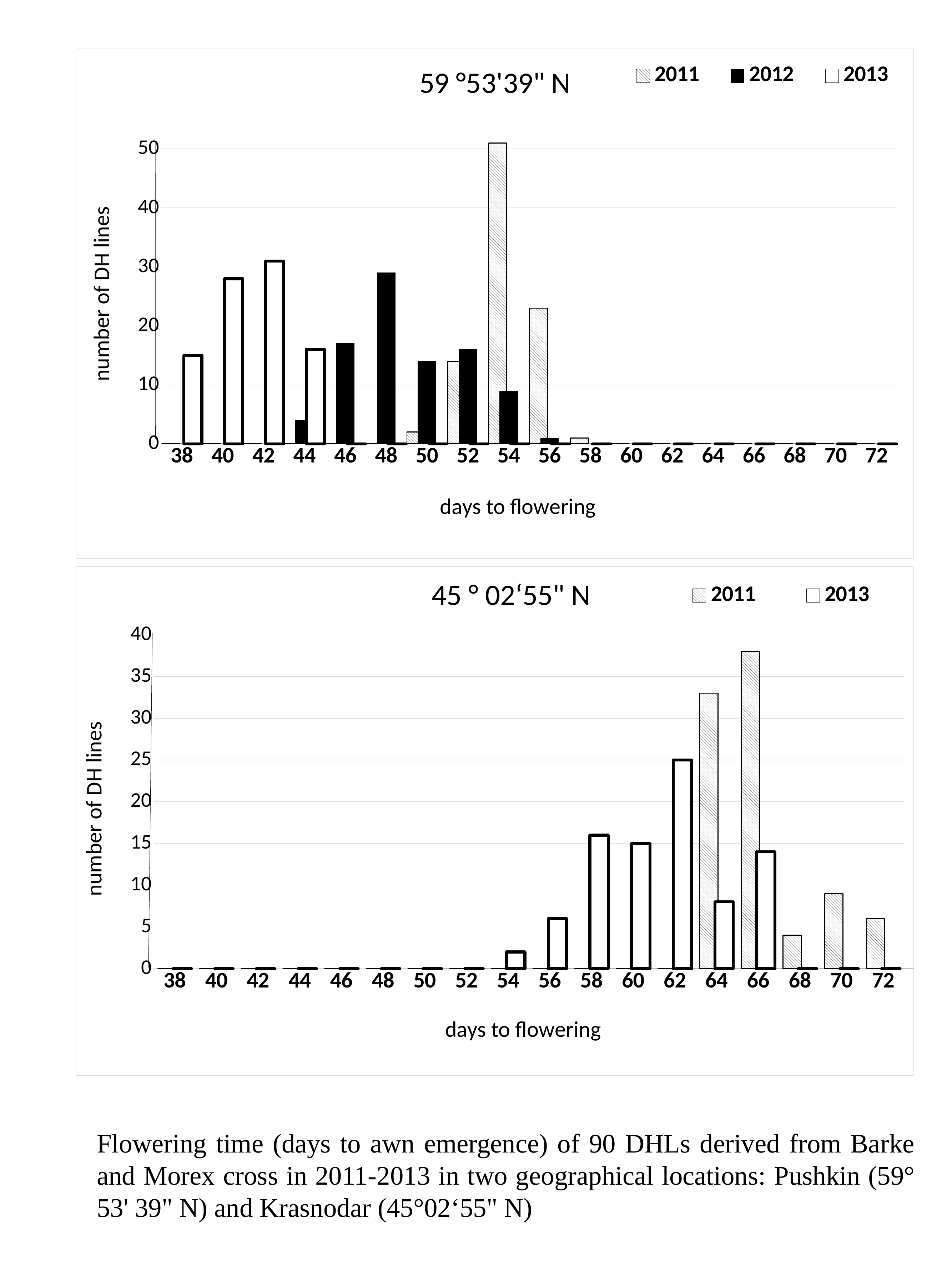

### Chart: 59 °53'39" N
| Category | | | |
|---|---|---|---|
| 38 | 0.0 | 0.0 | 15.0 |
| 40 | 0.0 | 0.0 | 28.0 |
| 42 | 0.0 | 0.0 | 31.0 |
| 44 | 0.0 | 4.0 | 16.0 |
| 46 | 0.0 | 17.0 | 0.0 |
| 48 | 0.0 | 29.0 | 0.0 |
| 50 | 2.0 | 14.0 | 0.0 |
| 52 | 14.0 | 16.0 | 0.0 |
| 54 | 51.0 | 9.0 | 0.0 |
| 56 | 23.0 | 1.0 | 0.0 |
| 58 | 1.0 | 0.0 | 0.0 |
| 60 | 0.0 | 0.0 | 0.0 |
| 62 | 0.0 | 0.0 | 0.0 |
| 64 | 0.0 | 0.0 | 0.0 |
| 66 | 0.0 | 0.0 | 0.0 |
| 68 | 0.0 | 0.0 | 0.0 |
| 70 | 0.0 | 0.0 | 0.0 |
| 72 | 0.0 | 0.0 | 0.0 |
### Chart: 45 ° 02‘55" N
| Category | | |
|---|---|---|
| 38 | 0.0 | 0.0 |
| 40 | 0.0 | 0.0 |
| 42 | 0.0 | 0.0 |
| 44 | 0.0 | 0.0 |
| 46 | 0.0 | 0.0 |
| 48 | 0.0 | 0.0 |
| 50 | 0.0 | 0.0 |
| 52 | 0.0 | 0.0 |
| 54 | 0.0 | 2.0 |
| 56 | 0.0 | 6.0 |
| 58 | 0.0 | 16.0 |
| 60 | 0.0 | 15.0 |
| 62 | 0.0 | 25.0 |
| 64 | 33.0 | 8.0 |
| 66 | 38.0 | 14.0 |
| 68 | 4.0 | 0.0 |
| 70 | 9.0 | 0.0 |
| 72 | 6.0 | 0.0 |Flowering time (days to awn emergence) of 90 DHLs derived from Barke and Morex cross in 2011-2013 in two geographical locations: Pushkin (59° 53' 39" N) and Krasnodar (45°02‘55" N)
